# A Parallel Thrombolysis Protocol with Nurse Practitioners As Coordinators Minimized Door-to-Needle Time for Acute Ischemic Stroke

**DOI:** 10.4061/2011/198518

**Published:** 2011-12-07

**Authors:** Sheng-Feng Sung, Ying-Chieh Huang, Cheung-Ter Ong, Yu-Wei Chen

**Affiliations:** ^1^Department of Neurology, Ditmanson Medical Foundation Chia-Yi Christian Hospital, Chiayi City 60002, Taiwan; ^2^Department of Emergency Medicine, Ditmanson Medical Foundation Chia-Yi Christian Hospital, Chiayi City 60002, Taiwan; ^3^Department of Neurology, Landseed Hospital, No. 77, Kwang-Tai Rd., Ping-Jen City 32449, Tao-Yuan County, Taiwan; ^4^Department of Neurology, National Taiwan University Hospital, No. 7, Chung Shan S. Rd., Taipei 100, Taiwan

## Abstract

*Introduction*. Quick thrombolysis after stroke improved clinical outcomes. The study objective was to shorten door-to-needle time for thrombolysis. *Methods*. After identifying the sources of in-hospital delays, we developed a protocol with a parallel algorithm and recruited nurse practitioners into the acute stroke team. We applied the new protocol on stroke patients from October 2009 to September 2010. Patients from the previous two years were used for comparison. *Results*. For ischemic stroke patients within 3 hours of onset, the median time from arrival to computed tomography scanning was reduced from 29 to 20 minutes (*P* < 0.001) and the median time from arrival to neurology evaluation decreased from 61 to 43 minutes (*P* < 0.001). For those patients who received thrombolysis, the median door-to-needle time was shortened from 68.5 to 58 minutes (*P* < 0.05). *Conclusions*. The parallel thrombolysis protocol successfully improved the median door-to-needle time to below the guideline-recommended 60 minutes.

## 1. Introduction

The introduction of intravenous recombinant tissue plasminogen activator (rtPA) has revolutionized the management of acute ischemic stroke (AIS). Treatment with rtPA has been shown to improve patients' outcomes at 3 months; however, its effectiveness decreased with time from the onset of stroke symptoms [1, 2]. Many stroke patients eligible for thrombolysis were not treated appropriately because of delayed presentation to the hospital or delayed examinations and management in the hospital. Although delays are mainly caused by patients themselves [3], it should be possible to minimize the in-hospital delay. According to the recommendations made by the National Institute of Neurological Disorders and Stroke, a patient with AIS should receive rtPA within 60 minutes of arrival at the emergency department (ED) [4]. A pilot study to address the quality of acute stroke care in 4 states of the US found that less than 20% of the patients treated with intravenous rtPA received it within 60 minutes of arrival [5]. A quasi-experimental trial (The Stroke Practice Improvement Network) to improve adherence to stroke performance measures concluded that the implementation of site-specific interventions did not increase the proportion of delivery of thrombolytic therapy within one hour of hospital arrival during the 6-month intervention period [6].

In Taiwan, only a minority of stroke patients are treated with rtPA. A nationwide study (Taiwan Stroke Registry) showed that 10.42% of AIS patients arriving within 2 hours of onset were treated with rtPA [7]. Although a study indicated that the adoption of less restrictive exclusion criteria for rtPA significantly increased the number of patients eligible for thrombolysis, there were still only 6.3% of patients who arrived within 3 hours of stroke onset received thrombolytic therapy [8]. Insufficient time to complete required studies was a main reason for exclusion from rtPA. Thus, this study was aimed to determine if the modification of protocol shortened the in-hospital delay and facilitated thrombolytic therapy.

## 2. Materials and Methods

This was a before-and-after study to investigate the effectiveness of implementation of a new thrombolysis protocol. Our institution is a 1000-bed community hospital serving a city and its adjoining rural area of around 500,000 inhabitants in southern Taiwan. The study population consisted of all AIS patients directly presenting to the ED within 3 hours of stroke onset in the one-year period from October 2009 to September 2010 (Period II) after the implementation of the new thrombolysis protocol. The major modification is that a nurse practitioner (NP) was designated to coordinate the newly designed parallel pathway for candidate patients. The control group comprised those patients who presented in the two-year period from October 2007 to September 2009 (Period I). The number of neurologists (five) on the acute stroke team and the number of computed tomography (CT) scanners (two) in the study hospital did not change during these two periods.

A standardized data abstraction form has been used in the registration of stroke patients in our institute since September 2006. We recorded the demographics, clinical characteristics, laboratory findings, radiological characteristics, and medications before and during hospitalization of the patients. The stroke severity was recorded on presentation by National Institute of Health Stroke Scale (NIHSS). The exact time of arrival at ED, evaluation by neurologists, receiving CT scans, and onset of thrombolysis were collected prospectively by a trained study nurse. The details in Taiwan Stroke Registry with the similar design had been described elsewhere [7]. The outcome recorded in this study included mortality and functional status at discharge, presented by modified Rankin Scale (mRS) score. The status at 3 months after discharge was obtained by the study nurse from the medical record or personal/telephone interview. The eligibility of thrombolysis for each patient was reviewed retrospectively by two senior neurologists according to the exclusion criteria set by the Department of Health and Bureau of National Health Insurance in Taiwan [8]. Every patient had a follow-up head CT scan 24 hours after thrombolytic therapy. To evaluate the safety of thrombolysis, we defined symptomatic intracranial hemorrhage (SICH) as any hemorrhage plus a neurological deterioration of 4 or more points on the NIHSS [9]. The data collection had been approved by the Institutional Review Board.

**Figure 1:**
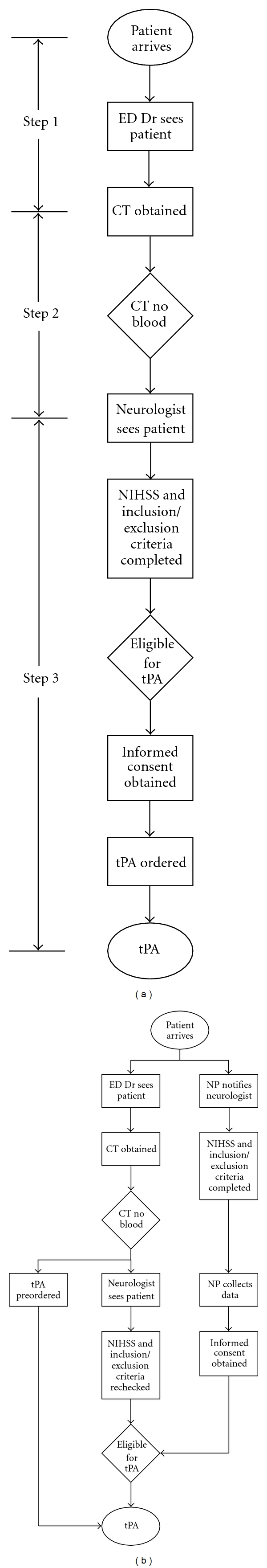
The original thrombolysis protocol based on a sequential algorithm (a) and the new thrombolysis protocol based on a parallel algorithm (b).

By analyzing our original thrombolysis protocol designed in 2007 (Figure 1(a)), we divided the door-to-needle time (DNT) into three steps: from ED arrival to CT scanning, from CT scanning to neurology evaluation, and from neurology evaluation to start of thrombolysis. The median time of obtaining a CT scan had been within 25 minutes before this study [10]. The major in-hospital delays occurred in the latter two steps. The prior protocol was an inefficient sequential algorithm (Figure 1(a)). Therefore, we implemented a parallel protocol (Figure 1(b)) to minimize the delays, and ED NPs were incorporated as coordinators into the acute stroke team to collaborate with the physicians and other departments.

We have a total of five NPs working morning and evening shifts in the ED. The ED NPs help take care of all emergency patients. However, once a patient was suspected to have AIS at the triage desk, an NP was assigned to this patient until rtPA was administered or the diagnosis was proven otherwise. If the patient was a candidate for thrombolysis based on the screening criteria, the designated NP would soon notify the on-call neurologist by telephone before the patient was sent for noncontrast CT scanning. In addition, the NP coordinated the patient care, including initial assessment of NIHSS and evaluation of suitability for thrombolysis, collections of CT images and results of laboratory tests, and preliminary explanation regarding the benefits and risks of thrombolytic therapy to the patients and/or their family. Hence multiple tasks can be done in parallel by the collaboration of the ED physician, the NP, the on-call neurologist, and other departments.

While the neurologist was evaluating a candidate patient for thrombolytic therapy, rtPA was brought to the bedside unmixed pending further treatment decision making. If the patient was determined eligible for thrombolysis, rtPA was administered immediately after informed written consent was obtained from the patient or next of kin. If thrombolysis was not indicated, the drug box was returned unopened to the pharmacy.

To investigate the effectiveness of the new protocol, we assessed time intervals between ED arrival and actions including: CT scan, reports of blood tests, neurology evaluation, and thrombolysis. To monitor the efficiency of thrombolysis over time, we computed the running median of DNTs at 3-month intervals in the control and study periods. The patients with mRS 0-1 were considered as having a favorable functional outcome.

Median values and interquartile ranges of the time intervals were used for descriptive statistics because of their nonnormal distributions. Comparison of median values was done with the Mann-Whitney test. Student's *t*-test was used to evaluate differences in continuous variables with normal distribution. Chi-square test or Fisher's exact test was used as appropriate to compare categorical data. A value of *P* < 0.05 was regarded as significant. All statistical analyses were performed using Windows SPSS version 15.0 (SPSS Inc., Chicago, ILL, USA).

## 3. Results

**Figure 2:**
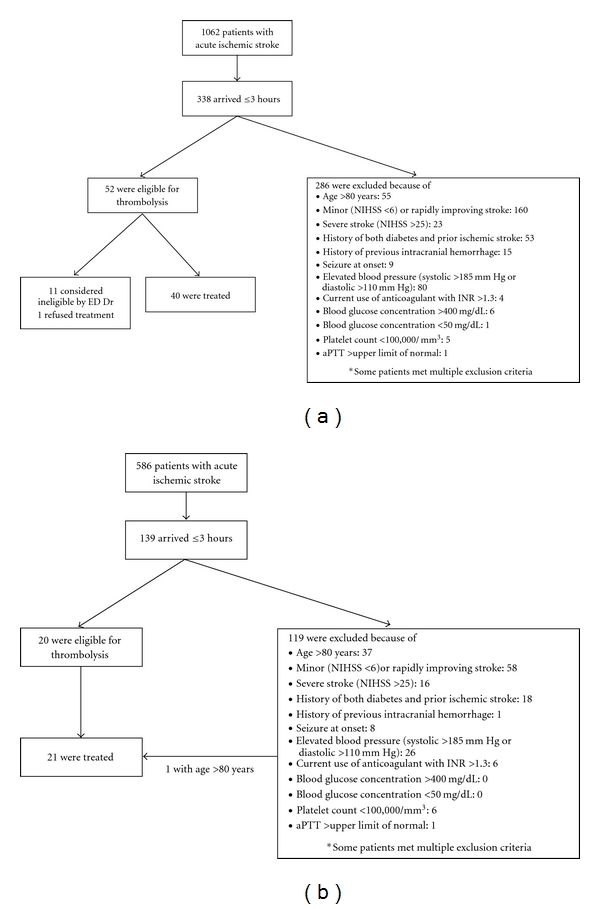
Clinical results before (a) and after (b) the implementation of the new thrombolysis protocol.

In Period I, a total of 1062 AIS patients were admitted, with 338 patients arriving within 3 hours. They were examined using the original thrombolysis protocol. Of these 338 patients, 52 (15.4%) patients were eligible for thrombolysis. In Period II, 586 patients with AIS were admitted and 139 of them arrived within 3 hours. Twenty (14.4%) patients were indicated to have thrombolysis. Common reasons for exclusion from treatment included age over 80 years, minor or rapidly improving stroke, severe stroke, history of both diabetes and prior ischemic stroke, and elevated blood pressure (Figure 2). During Period I, one patient refused thrombolysis and 11 patients were either considered ineligible by ED doctors or were unable to complete CT and laboratory studies in time. During Period II, in addition to the 20 eligible patients, one patient who met the exclusion criteria because of age was treated as per family request. Therefore, for patients within 3 hours of onset, the treatment rate increased from 11.8% (40/338) to 15.1% (21/139) between Periods I and II (Table 1). There was no significant difference in the proportion of eligible patients.

**Figure 3:**
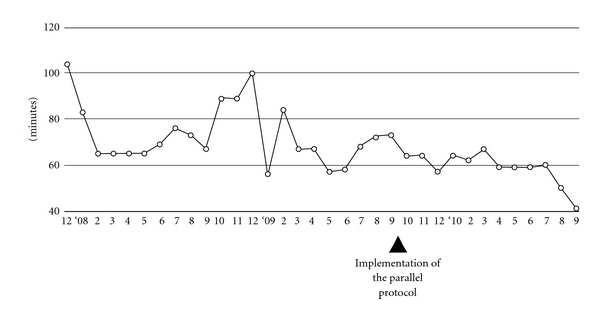
Time series of the three-month running median of door-to-needle times.

 For all the patients with AIS directly presenting to the ED within 3 hours of stroke onset, the median time from arrival to CT examination decreased significantly from 29 to 20 minutes. At the same time, the median time from arrival to neurology evaluation was reduced considerably from 61 to 43 minutes (Table 1). For those patients treated with rtPA, the implementation of the new protocol significantly reduced the door-to-neurology evaluation time from 46 to 37 minutes and the DNT from 68.5 to 58 minutes (Table 2). The 3-month running median of DNTs also decreased from 104 to 40 minutes over the 3-year period (Figure 3). The onset-to-needle time was not changed despite longer onset-to-door time during Period II, reflecting that we administered rtPA to more patients with delayed presentation of more than 2 hours after onset (19% in Period II versus none in Period I). This factor also contributes to a nonsignificant increase in the proportion of thrombolysed patients. The time from arrival to report of prothrombin time/partial thromboplastin time, remained around 50 minutes. Baseline NIHSS scores and the proportion of patients with favorable outcome after 3 months did not change between the two periods.

## 4. Discussion

We demonstrated the significant effect on shortening DNT of rtPA administration in this before-and-after comparison in the same hospital. The major changes between the two periods are the implementation of the parallel protocol and the introduction of NPs as coordinators in ED. Since intravenous rtPA should be administered within the narrow 3-hour time window, a substantial proportion of patients who arrived early did not receive thrombolytic therapy owing to in-hospital delays including: delayed physician evaluation, neurologic consultation, neuroimaging, and laboratory tests [11]. In-hospital delays can be shortened through the organization of a stroke team, the development of stroke pathways, and the training of ED personnel [12]. Although recent studies proved that rtPA is effective up to 4.5 hours after stroke onset [13], its effectiveness decays with the time between onset and treatment [2]. Therefore, every effort should be taken to hasten the start of the treatment, and the target treatment with rtPA should be within one hour of patient's arrival in the ED [14].

By using a parallel strategy to overcome the in-hospital delays, our revised protocol effectively reduced the median time of DNT below the recommended 60 minutes. Rapid identification of potential candidates for thrombolysis is the paramount first step in order that neurology evaluation, CT scans, and laboratory studies can be arranged immediately upon ED arrival. However, this poses a challenge to busy ED physicians. In Period II, an NP was soon assigned to a thrombolytic candidate upon hospital arrival. After preliminary screening, the NP notified the neurologist who made the decision of thrombolysis. This practice could prevent a busy ED physician from excluding patients who actually qualify for treatment because of delayed examinations or unfamiliarity with rtPA eligibility criteria, as we have shown that 21% (11/52) of eligible patients were not treated in Period I but all eligible patients were treated in Period II. In one study, although the agreement for determination of rtPA eligibility was good between ED attendings and stroke neurologists, 18% of thrombolysis candidates were still designated as ineligible by ED attendings and the proportion was even higher for ED residents [15].

Physicians who are not neurologists tend not to give patients thrombolytic treatment [16]. ED physicians usually did not administer rtPA until a neurologist was called in for consultation, considering the high rate of SICH among the Chinese-Taiwanese [17]. Because of the limited personnel and funding resources in a community hospital setting, neurologists are not available on a 24/7 basis at our institution. Since a team approach is important for the successful implementation of a stroke protocol [18] and NP care has been shown to increase compliance with clinical practice guidelines [19], we incorporated ED NPs into our acute stroke tame. The Calgary Stroke Program has demonstrated that the use of stroke NPs reduced the DNT from 90 to 60 minutes and the door-to-CT time from 60 to 30 minutes [20]. Nonetheless, the role of NPs in our stroke team is different from that in the Calgary Stroke Program in two aspects. First, the NPs are not dedicated stroke NPs and they also carry out routine jobs in the ED. Second, they coordinate and facilitate the whole care process, but not as autonomous ED care providers [21]. The obvious drawbacks are as follows: the neurologists still need to assess the patients in person, and the NPs have to defer their work when a stroke patient arrives. However, this has the advantage of not increasing direct hospital costs.

Thrombolytic therapy inevitably carries the risk of SICH, especially when there is any deviation from the preset criteria [22, 23]. Hence stroke patients must be checked against a lengthy list of exclusion criteria which require a detailed time-consuming history taking. For this purpose, the NP helped complete the exclusion checklist while the neurologist was on the way to the bedside. It could be a concern if shortening of DNT might cause some patients to be thrombolysed without fulfilling strict criteria. In the present study, the reduction in DNT was not associated with an increased proportion of SICH and all the thrombolysed patients were eligible except one patient aged over 80 years. Furthermore, our revised protocol may prevent inappropriate use of rtPA because the exclusion criteria were checked by both the NP and the neurologist. Another concern regarding our practice is that we do not wait for formal CT interpretation by radiologists. Such practice seemed to be safe and did not increase the risks of SICH [24].

The introduction of a computerized in-hospital alert system has significantly reduced the time intervals from ED arrival to evaluation steps and treatment [25]. However, hospitals have to be equipped with computerized network systems and have to develop the computer program. By adoption of a parallel algorithm and recruitment of NPs into the acute stroke team, our thrombolysis protocol has been a success similar to the computerized system.

Although the new protocol has shortened the DNT below 60 minutes, it is far from satisfactory because ultraearly thrombolysis (treatment within 70 minutes of stroke onset) resulted in a much higher likelihood of good outcomes for patients with moderate and severe strokes [26]. A recent study has demonstrated that the median DNT could be decreased to only 20 minutes in a university hospital setting [27]. In the present study, the time from arrival to availability of coagulation tests was not improved significantly. Therefore the time spent in the central laboratory blood analysis will be a main limiting factor for further reduction of DNT. One approach is to proceed to treatment pending the results of prothrombin time or partial thromboplastin time unless there is a clinical reason to expect abnormal results of these tests [24]. Another approach is to use point-of-care devices for measurements of international normalized ratio for patients taking oral anticoagulants or when information regarding anticoagulation is unavailable. The use of point-of-care device (Coaguchek XS; Roche, Switzerland) saved an average of 28 minutes in one study [28].

Despite the successful shortening of DNT in this study, the percentage of stroke patients treated with rtPA is still small. To overcome this problem, community programs to educate patients to seek treatment sooner after a stroke should be an integral component of stroke care. In addition, an effective prehospital stroke code system should be established. Prehospital notification by EMS not only shortened the prehospital delay [29], but also reduced DNT [30, 31]. The prenotification ensures that the CT is ready and available for the arriving stroke patient and makes it possible for the stroke neurologist to already be at the ED when the patient arrives. The combined effect of shortening prehospital delay and in-hospital delay would further decrease the treatment time from symptom onset, resulting in improved patients' outcomes.

This study does have several limitations. First, we did not have statistical power to detect a difference in the rates of thrombolysis between the two periods. However, we did demonstrate that shortening of in-hospital delays might increase the number of thrombolysed patients, especially for those who arrived more than two hours after onset. Inclusion of more patients with late arrivals might explain the lack of a decrease in onset-to-needle time in the study period. Second, the improvement in DNT might be partly due to the continuing education and training of the medical staff. Third, we could not show an improvement in the 3-month outcomes of patients. It takes many factors to achieve favorable functional outcomes, including shortened onset-to-needle time. The combined effect of decreased prehospital and in-hospital delays can be explored in future studies.

## 5. Conclusions

It is possible to shorten the time intervals of stroke management by assigning an NP as an ED coordinator of the parallel thrombolysis protocol to overcome the specific sources of delays in a community hospital setting with limited resources and faculty. Our successful model may help to promote the efficient use of NPs in team-based care for acute stroke patients.

## Figures and Tables

**Table 1:** Patients with acute ischemic stroke directly presenting to the emergency department within 3 hours of stroke onset.

	2007/10–2009/9 (*n* = 338)	2009/10–2010/9 (*n* = 139)	*P* value
Age, mean ± SD: y	69.1 ± 12.5	71.9 ± 12.5	0.029
Female, *n* (%)	136 (40.2)	59 (42.4)	0.656
Time from onset to, median (IQR): min			
Arrival	65 (34–108)	66 (36–117)	0.217
Time from arrival to, median (IQR): min			
CT scan	29 (19–50)	20 (13–38)	<0.001
Neurology evaluation	61 (40–96)	43 (31–61)	<0.001
Eligible for rtPA, *n* (%)	52 (15.4)	20 (14.4)	0.782
Treated with rtPA, *n* (%)	40 (11.8)	21 (15.1)	0.331

CT: computed tomography; IQR: interquartile range; SD: standard deviation; rtPA: recombinant tissue plasminogen activator.

**Table 2:** Patients treated with thrombolytic therapy.

	2007/10–2009/9 (*n* = 40)	2009/10–2010/9 (*n* = 21)	*P* value
Age, mean ± SD: y	65.6 ± 12.1	71.3 ± 13.3	0.095
Female, *n* (%)	12 (30.0)	9 (42.9)	0.315
Pretreatment NIHSS, median	16	18	0.451
Time from onset to, median (IQR): min			
Arrival	38.5 (22–73)	54 (27–103)	0.151
Thrombolysis	112.5 (95–137)	121 (88–158)	0.796
Arrival between 2 and 3 hours, *n* (%)	0 (0)	4 (19.0)	0.012
Time from arrival to, median (IQR): min			
CT scan	16.5 (12–23)	14 (11–19)	0.248
PT/PTT	52 (46–58)	48 (39–60)	0.288
Neurology evaluation	46 (32–63)	37 (28–43)	0.026
Thrombolysis	68.5 (57–83)	58 (54–69)	0.035
ICU admission	133.5 (95–152)	116 (94–143)	0.230
mRS 0-1, *n *(%)	14 (35.0)	6 (28.6)	0.611
SICH, *n* (%)	5 (12.5)	2 (9.5)	1.000^a^

^a^Fisher's exact test.

CT: computed tomography; ICU: intensive care unit; IQR: interquartile range; mRS: modified Rankin Scale; NIHSS: National Institutes of Health Stroke Scale; PT: prothrombin time; PTT: partial thromboplastin time; SD: standard deviation; SICH: symptomatic intracerebral hemorrhage; rtPA: recombinant tissue plasminogen activator.

## References

[1] Marler JR, Tilley BC, Lu M (2000). Early stroke treatment associated with better outcome: the NINDS rt-PA stroke study. *Neurology*.

[2] Lees KR, Bluhmki E, von Kummer R (2010). Time to treatment with intravenous alteplase and outcome in stroke: an updated pooled analysis of ECASS, ATLANTIS, NINDS, and EPITHET trials. *The Lancet*.

[3] Boode B, Welzen V, Franke C, van Oostenbrugge R (2007). Estimating the number of stroke patients eligible for thrombolytic treatment if delay could be avoided. *Cerebrovascular Diseases*.

[4] Marler JR, Jones PW, Emr M Setting new directions for stroke care.

[5] Reeves MJ, Arora S, Broderick JP (2005). Acute stroke care in the US: results from 4 pilot prototypes of the Paul Coverdell national acute stroke registry. *Stroke*.

[6] Hinchey JA, Shephard T, Tonn ST (2010). Stroke practice improvement network: a quasiexperimental trial of a multifaceted intervention to improve quality. *Journal of Stroke and Cerebrovascular Diseases*.

[7] Hsieh FI, Lien LM, Chen ST (2010). Get with the guidelines-stroke performance indicators: surveillance of stroke care in the Taiwan stroke registry: get with the guidelines-stroke in Taiwan. *Circulation*.

[8] Huang P, Khor GT, Chen CH, Lin RT, Liu CK (2011). Eligibility and rate of treatment for recombinant tissue plasminogen activator in acute ischemic stroke using different criteria. *Academic Emergency Medicine*.

[9] Hacke W, Kaste M, Fieschi C (1998). Randomised double-blind placebo-controlled trial of thrombolytic therapy with intravenous alteplase in acute ischaemic stroke (ECASS II). *The Lancet*.

[10] Sung SF, Ong CT, Wu CS, Hsu YC, Su YH (2010). Increased use of thrombolytic therapy and shortening of in-hospital delays following acute ischemic stroke: experience on the establishment of a primary stroke center at a community hospital. *Acta Neurologica Taiwanica*.

[11] Cocho D, Belvís R, Martí-Fàbregas J (2005). Reasons for exclusion from thrombolytic therapy following acute ischemic stroke. *Neurology*.

[12] Chen CH, Huang P, Yang YH, Liu CK, Lin TJ, Lin RT (2007). Pre-hospital and in-hospital delays after onset of acute ischemic stroke: a hospital-based study in southern Taiwan. *Kaohsiung Journal of Medical Sciences*.

[13] Hacke W, Kaste M, Bluhmki E (2008). Thrombolysis with alteplase 3 to 4.5 hours after acute ischemic stroke. *The New England Journal of Medicine*.

[14] Summers D, Leonard A, Wentworth D (2009). Comprehensive overview of nursing and interdisciplinary care of the acute ischemic stroke patient: a scientific statement from the American heart association. *Stroke*.

[15] Mecozzi AC, Brown DL, Lisabeth LD (2007). Determining intravenous rt-PA eligibility in the emergency department. *Neurocritical Care*.

[16] Reed SD, Cramer SC, Blough DK, Meyer K, Jarvik JG (2001). Treatment with tissue plasminogen activator and inpatient mortality rates for patients with ischemic stroke treated in community hospitals. *Stroke*.

[17] Chao AC, Hsu HY, Chung CP (2010). Outcomes of thrombolytic therapy for acute ischemic stroke in Chinese patients: the Taiwan thrombolytic therapy for acute ischemic stroke (TTT-AIS) study. *Stroke*.

[18] Gil Núñez AC, Mora JV (2004). Organization of medical care in acute stroke: importance of a good network. *Cerebrovascular Diseases*.

[19] Kleinpell RM, Ely EW, Grabenkort R (2008). Nurse practitioners and physician assistants in the intensive care unit: an evidence-based review. *Critical Care Medicine*.

[20] Green T, Newcommon N (2006). Advancing nursing practice: the role of the nurse practitioner in an acute stroke program. *The Journal of Neuroscience Nursing*.

[21] Steiner IP, Blitz S, Nichols DN, Harley DD, Sharma L, Stagg AP (2008). Introducing a nurse practitioner into an urban Canadian emergency department. *Canadian Journal of Emergency Medicine*.

[22] Katzan IL, Furlan AJ, Lloyd LE (2000). Use of tissue-type plasminogen activator for acute ischemic stroke: the cleveland area experience. *Journal of the American Medical Association*.

[23] Lopez-Yunez AM, Bruno A, Williams LS, Yilmaz E, Zurrú C, Biller J (2001). Protocol violations in community-based rTPA stroke treatment are associated with symptomatic intracerebral hemorrhage. *Stroke*.

[24] Sattin JA, Olson SE, Liu L, Raman R, Lyden PD (2006). An expedited code stroke protocol is feasible and safe. *Stroke*.

[25] Heo JH, Kim YD, Nam HS (2010). A computerized in-hospital alert system for thrombolysis in acute stroke. *Stroke*.

[26] Strbian D, Soinne L, Sairanen T (2010). Ultraearly thrombolysis in acute ischemic stroke is associated with better outcome and lower mortality. *Stroke*.

[27] Mustanoja S, Meretoja A, Putaala J (2011). Outcome by stroke etiology in patients receiving thrombolytic treatment: descriptive subtype analysis. *Stroke*.

[28] Rizos T, Herweh C, Jenetzky E (2009). Point-of-care international normalized ratio testing accelerates thrombolysis in patients with acute ischemic stroke using oral anticoagulants. *Stroke*.

[29] Morris DL, Rosamond W, Madden K, Schultz C, Hamilton S (2000). Prehospital and emergency department delays after acute stroke: the genentech stroke presentation survey. *Stroke*.

[30] Lindsberg PJ, Häppölä O, Kallela M, Valanne L, Kuisma M, Kaste M (2006). Door to thrombolysis: ER reorganization and reduced delays to acute stroke treatment. *Neurology*.

[31] Kim SK, Lee SY, Bae HJ (2009). Pre-hospital notification reduced the door-to-needle time for iv t-PA in acute ischaemic stroke. *European Journal of Neurology*.

